# Variation of Ultrasound Findings in the First Trimester Examination of Recurrent Cases With Trisomy 21

**DOI:** 10.14740/jocmr2138w

**Published:** 2015-04-08

**Authors:** Aggelos Daniilidis, Dimitrios Balaouras, Dimitrios Chitzios, Georgios Balaouras, Mihai Capilna, Efstratios Asimakopoulos

**Affiliations:** a The 2nd University Department of Obstetrics and Gynecology, Hippokratio General Hospital, Aristotle University of Thessaloniki, Konstantinoupoleos 49, 54642 Thessaloniki, Greece; b Department of Medical Biopathology, University Hospital of Thessalia, Larisa, Greece; c University Department of Obstetrics and Gynecology, Tirgu Mures, Romania

**Keywords:** Nuchal translucency, Hydrops fetalis, Encephalocele, Congenital diseases, Fetal ultrasound scan, Serum screening

## Abstract

Increased nuchal translucency (NT) is present in about 50% of cases with trisomy 21. Very often the nuchal edema evolves in hydrops fetalis until the second trimester. Furthermore, a small amount of cases with a normal NT and trisomy 21 exhibit anatomical anomalies. We present a case of a 21-year-old woman, nulliparous, with a history of one termination of pregnancy and a smoking quitter. The prenatal control was negative for TORCH. During the first trimester scan on the 13th week, the NT was found 2.7 mm, the ductus venosus Doppler was normal, and the nasal bone was present. Hydrops fetalis was present though, and the parents were advised for chorionic villus sampling (CVS), but they opted for termination of pregnancy. The molecular control by QF-PCR showed normal karyotype for 13 and 18, a male fetus, but non-dysjunction trisomy 21 was present. Parental karyotype was advised, but they refused to perform it. One year later, the couple had another pregnancy. On the 12th week scan, the NT was found 1.0 mm, the ductus venosus Doppler was normal, and the nasal bone was present, but encephalocele was also found, and the parents consented again for termination of pregnancy. The new molecular control showed the same results. This time parental karyotype was performed. The father had a normal one, whereas the mother showed reversed p11 and q13 zones in chromosome 2. Genetical consulting and prenatal cytological control was advised in before next pregnancy.

## Introduction

Increased nuchal translucency (NT) is present in about 50% of cases with trisomy 21. Very often the nuchal edema evolves in hydrops fetalis until the second trimester. Furthermore, a small amount of cases with a normal NT and trisomy 21 exhibit anatomical anomalies [1, 2]. Human cells should contain 23 pairs of chromosomes. One chromosome in each pair comes from the father, the other from the mother. Down syndrome could be caused when abnormal cell division involving chromosome 21 recurs. These division abnormalities result in extra genetic material from chromosome 21, which is responsible for the characteristic features and developmental problems of Down syndrome. Any one of three genetic variations can cause Down syndrome, like trisomy 21 or translocation Down syndrome or mosaic Down syndrome [3]. About 95% of the time, Down syndrome is caused by trisomy 21 and the child has three copies of chromosome 21 in all cells. This is caused by abnormal cell division during the development of the sperm cell or the egg cell. Most of the time, Down syndrome is not inherited. It is caused by a mistake in cell division during the development of the egg, sperm or embryo. Translocation Down syndrome is the only form of the disorder that could be inherited from parent to child and about 4% of children with Down syndrome have translocation [4-6]. The 30% almost of these children inherited it from one of their parents. In mosaic Down syndrome, which is a rare, the children have some cells with an extra copy of chromosome 21. This mosaic of normal and abnormal cells is caused by abnormal cell division after fertilization [7, 8]. We present an interesting case of a couple in their first pregnancy with a fetus with increased NT, hydrops fetalis and trisomy 21 in karyotype, diagnosed by ultrasound at 13 weeks of gestation and in their second pregnancy with a fetus with normal NT, encephalocele and trisomy 21 again diagnosed by ultrasound and karyotyping at 12 weeks of gestation. We also present a brief review of the literature about occurrence, follow-up and diagnosis of increased NT, abnormal karyotype and isolated structural fetal abnormalities.

## Case Report

**Figure 1:**
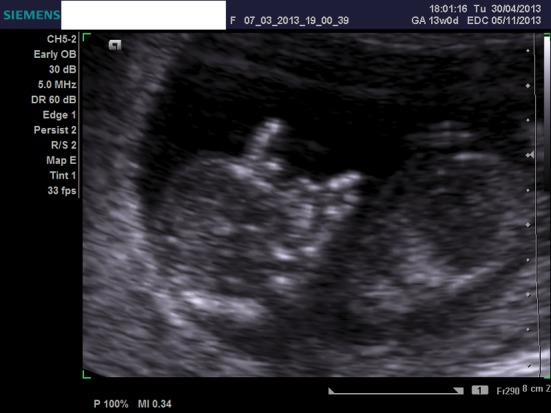
Ultrasound image of the second fetus at 12 weeks, showing the nuchal translucency and present nasal bone.

**Figure 2:**
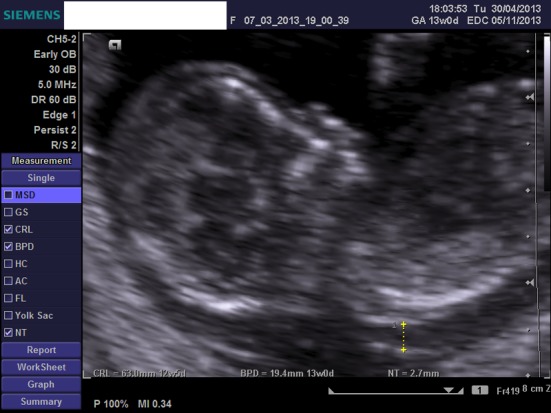
Ultrasound image at 12 weeks, showing the ductus venosus Doppler.

**Figure 3:**
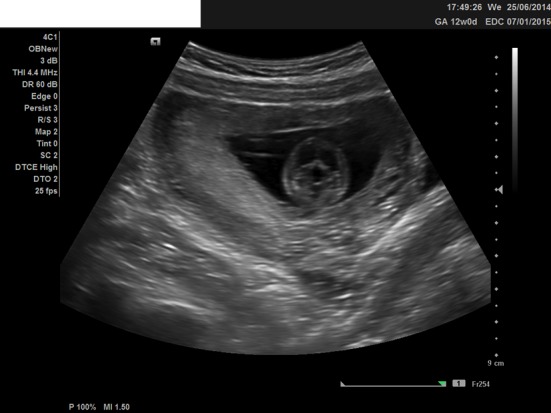
Ultrasound image at 12 weeks, showing the encephalocele, transverse view.

**Figure 4:**
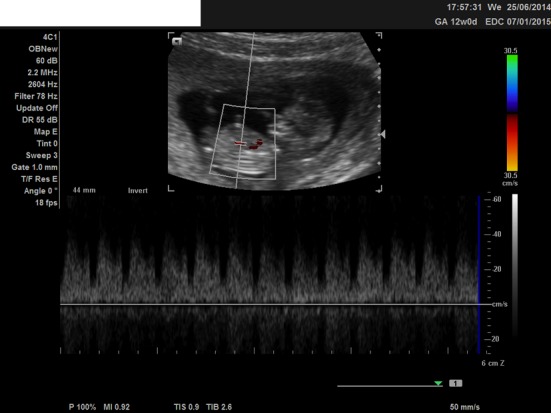
Ultrasound image of the first fetus at 13 weeks, showing the nuchal translucency.

**Figure 5:**
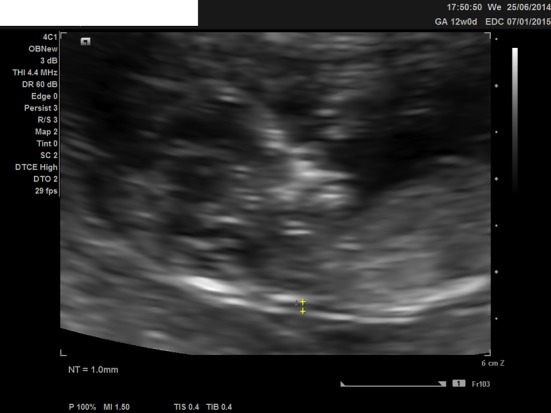
Ultrasound image at 13 weeks, showing the hydrops fetalis.

We present a case of a 21-year-old woman, nulliparous, with a history of one termination of pregnancy and a smoking quitter. The prenatal control was negative for TORCH and she was 0+ blood group. During the first trimester scan on the 13th week, the NT was found 2.7 mm, the ductus venosus Doppler was normal, and the nasal bone was present. Hydrops fetalis was present though, and the parents were advised for chorionic villus sampling (CVS), but they opted for termination of pregnancy (Fig. 1, 2). The molecular control by QF-PCR showed normal karyotype for 13 and 18, a male fetus, but non-dysjunction trisomy 21 was present. Parental karyotype was advised, but they refused to perform it. One year later, the couple had another pregnancy. On the 12th week scan, the NT was measured 1.0 mm, the ductus venosus Doppler was normal, the nasal bone was present, but encephalocele was also found (Fig. 3-5), and the parents were consented again for termination of pregnancy. The molecular control showed the same results. This time parental karyotype was performed. The father had a normal one, whereas the mother showed reversed p11 and q13 zones in chromosome 2. Genetical consulting and prenatal cytological control was advised in before next pregnancy.

## Discussion

Ongoing technological development, including high-frequency transvaginal scanning, has allowed the resolution of ultrasound imaging in the first trimester to a step at which early fetal development could be monitored in detail [9, 10]. First trimester echo here refers to a stage of pregnancy starting from the time at which viability can be confirmed which means the presence of a gestational sac in the uterus with an embryo reporting cardiac activity up to 13 + 6 weeks of gestation [11-14]. Towards the end of the first trimester, the echo could give an opportunity to detect gross fetal abnormalities and it offers first-trimester aneuploidy screening, measure the NT thickness [15, 16]. However we should refer that many malformations may develop later in pregnancy or may not be detected even with appropriate equipment and in the most experienced of hands [17]. The second-trimester 18 - to 22-week echo remains the standard for the detection of fetal anatomy in both low-risk and high-risk pregnancies [18].

First-trimester evaluation of fetal anatomy and detection of anomalies was introduced in the late 1980s and early 1990s with the advent of effective transvaginal probes. The introduction of NT aneuploidy screening in the 11- to 13 + 6-week window has given an interest in early anatomy scanning. The advantages include early detection and exclusion of many abnormalities, early reassurance to high-risk mothers, earlier genetic diagnosis and easier pregnancy termination if appropriate. Limitations include trained and experienced personnel, uncertain cost and late development of some anatomical structures and pathologies (e.g. corpus callosum, hypoplastic left heart), which make early detection impossible and can lead to difficulties in counseling [19].

The first-trimester screening should include NT measurement and in addition of some other markers, including biochemical measurement of free beta or total human chorionic gonadotropin (hCG) and pregnancy-associated plasma protein-A (PAPP-A) [20]. In appropriate circumstances, additional aneuploidy markers, including nasal bone, tricuspid regurgitation, ductal regurgitation and some others, may be sought [21, 22]. It is recommended that NT should be measured between 11 and 13 + 6weeks, corresponding to a CRL measurement of between 45 and 84 mm. This gestational age window is chosen because NT as a screening test performs optimally and fetal size allows diagnosis of major fetal abnormalities, which can provide to women who are carrying an affected fetus with the option of an early termination of pregnancy [23].

### Conclusion

The sensitivity of NT for trisomy 21 is about 80% for the 5% of the general population, considered as in high risk. The increased NT is also associated with some other chromosomal anomalies and its sensitivity is 75% for trisomy 18, 72% for trisomy 13 and 87% for Turner’s syndrome. The fetuses with increased NT show increased endometrial morbidity and increased risk for cardiac anomalies or genetic syndromes [1, 8, 9].
